# Glass transition temperatures and crystallization kinetics of a synthetic, anhydrous, amorphous calcium-magnesium carbonate

**DOI:** 10.1098/rsta.2022.0356

**Published:** 2023-10-16

**Authors:** Kai-Uwe Hess, Jürgen E. K. Schawe, Martin Wilding, Bettina Purgstaller, Katja E. Goetschl, Sebastian Sturm, Knut Müller-Caspary, Elena V. Sturm, Wolfgang Schmahl, Erika Griesshaber, Thilo Bissbort, Daniel Weidendorfer, Martin Dietzel, Donald B. Dingwell

**Affiliations:** ^1^ Earth and Environmental Sciences, Ludwig-Maximilians-Universität München, Theresienstraße 41/III, 80333 München, Germany; ^2^ Mettler-Toledo GmbH, Heuwinkelstrasse 3, 8603, Nänikon, Switzerland; ^3^ Laboratory of Metal Physics and Technology, Department of Materials, ETH Zurich, 8093 Zurich, Switzerland; ^4^ UK Catalysis Hub, Research Complex at Harwell, Rutherford Appleton Laboratory, Harwell Campus, Oxfordshire OX11 0FA, UK; ^5^ Institute of Applied Geosciences, Graz University of Technology, Rechbauerstrasse 12, 8010 Graz, Austria; ^6^ Fakultät für Chemie und Pharmazie, Physikalische Chemie, Ludwig-Maximilians-Universität München, Butenandstr. 5-13, 81377, München, Germany

**Keywords:** amorphous calcium-magnesium carbonate, glass transition temperature, lyophilization, crystallization dynamics, scanning transmission electron microscopy, fast scanning differential scanning calorimetry

## Abstract

We report the first calorimetric observations of glass transition temperatures and crystallization rates of anhydrous, amorphous calcium-magnesium carbonate using fast scanning differential scanning calorimetry. Hydrous amorphous Ca_0.95_Mg_0.05_CO_3_ · 0.5H_2_O (ACMC) solid was precipitated from a MgCl_2_–NaHCO_3_ buffered solution, separated from the supernatant, and freeze-dried. An aliquot of the freeze-dried samples was additionally dried at 250°C for up to 6 h in a furnace and in a high-purity N_2_ atmosphere to produce anhydrous ACMC. The glass transition temperature of the anhydrous Ca_0.95_Mg_0.05_CO_3_ was determined by applying different heating rates (1000–6000 K s^−1^) and correcting for thermal lag to be 376°C and the relaxational heat capacity was determined to be *C*p = 0.16 J/(g K). Additionally, the heating rate dependence of the temperature that is associated with the corrected crystallization peaks is used to determine the activation energy of crystallization to be 275 kJ mol^−1^. A high-resolution transmission electron microscopy study on the hydrous and anhydrous samples provided further constraints on their compositional and structural states.

This article is part of the theme issue 'Exploring the length scales, timescales and chemistry of challenging materials (Part 1)'.

## 1. Introduction

Although carbonates occur throughout the geological record, amorphous forms are generally thought to be rare. Carbonate glasses do not occur naturally, although they can be synthesized (see Weidendorfer *et al*. [1]). By far the most common, naturally occurring forms of amorphous carbonates are those considered to be amorphous solids which are mainly, but not exclusively, produced by biogenic processes [2]. These materials contain large amounts of structural water and apparently different amorphous structures and show a complex path towards the crystallization of stable carbonate polymorphs involving dehydration steps and formation of intermediate, transient amorphous forms [3]. One key question is whether these amorphous forms can be distinguished from glasses, including carbonate glasses, that are formed by more conventional methods and whether the crystallization kinetics are determined by the same glassy dynamics that influence the devitrification of more traditional glasses.

In the broadest sense, a glass is defined as a condensed state of matter that has become non-ergodic [4]. This definition is derived from classical or structural glasses but can be applied to a range of materials that can be produced by different routes including glasses of interest to the food and pharmaceutical industry. This general definition of a glass requires that the material has some degree of freedom that fluctuates with temperature or pressure when the system is in equilibrium (ergodic) and can explore all states. When temperature or pressure are reduced then the fluctuations become frozen and these states can no longer be explored on the observational time scale and the system becomes non-ergodic, or glassy [5]. A structural glass is generally produced by cooling of liquids with an increase in viscosity and therefore structural relaxation time such that the liquid falls out of equilibrium and becomes non-ergodic over a range of temperature, the glass transformation range. This is accompanied by a change in heat capacity and on reheating of the glass there is relaxation to a metastable (ergodic), supercooled liquid with the relaxation of structure over this transformation range shown by the glass transition peak, characterized by the relaxation time and prior cooling rate. This definition of a structural glass can exclude metallic glasses, which would be defined as amorphous solids since many do not show a glass transition or jump in heat capacity but crystallize on reheating. This would have also relegated obviously materials such as forsterite (Mg_2_SiO_4_) glasses produced by rapid quenching of levitated drops to an amorphous material since in this case too crystallization occurs without observation of a glass transition [6]. A simple explanation for both metallic glasses and highly refractory silicate glasses such as forsterite is that the jump in heat capacity is obscured by the crystallization exotherm. Subsequently, expansion of the range of composition of metallic glass formation and faster scanning rates for DSC measurement for forsterite have identified more recognizable glass transitions.

With the definition of a glass transition as a transition from non-ergodic to ergodic behaviour there is an opportunity to widen the definition of glasses to include amorphous solids that are not formed by the conventional routes and to explore these materials by thermal analysis. There is potential to access the thermal signature of the glass transformation range by using very fast scanning rates.

Fast scanning differential scanning calorimetry (FDSC) based on MEMS chip sensors enables heating and cooling rates of several thousands of Kelvin per second [7,8]. The glass transition of Ca_0.95_Mg_0.05_CO_3_ · 0.5H_2_O cannot be measured by conventional DSC because the primary crystallization completely intervenes and its corresponding calorimetric signal overlays that of the glass transition. Due to the different heating rate dependencies (activation energies) of vitrification versus crystallization, it should be possible to separate the calorimetric signals of the two processes using higher scanning rates (compared to conventional DSC). In this manner the glass transition may be made accessible as measured by FDSC. Here we detail a successful application of FDSC to the determination of the glass transition of nominally anhydrous Ca_0.95_Mg_0.05_CO_3_.

## 2. Material and methods

### (a) Synthesis of amorphous calcium magnesium carbonate

The synthesis of hydrous amorphous calcium-magnesium carbonate (ACMC) was carried out according to a previously developed protocol [9,10]. Briefly, a 0.25 M (Ca, Mg)Cl_2_ solution and a 0.25 M Na_2_CO_3_ solution were prepared using CaCl_2_ 2H_2_O, MgCl_2_ 6H_2_O and Na_2_CO_3_ (Carl Roth chemicals) and ultrapure deionized water (Millipore Integrals 3; 18.2 MΩ cm^−1^). The relative Mg content of the stock solution ([Mg]_stock_ = {[Mg]/([Ca] + [Mg])} · 100 in %) was initially 12 mol% to yield 5 mol% Mg content in the solid according to the eq. [Mg]_ACMC_ = −21.03243 · {1 − exp(0.0175 · [Mg]_stock_)} (from [10]). The two stock solutions were precooled in a refrigerator for at least 4 h (T = 10 ± 1°C). Precipitation of ACMC was initiated by pouring 80 ml of the (Ca, Mg)Cl_2_ solution into a glass beaker containing 80 ml of Na_2_CO_3_ solution. The precipitated ACMC was immediately separated from the supernatant with a 0.2 mm cellulose filter using a suction filtration unit and transferred into a glass flask connected to a freeze-dryer (Virtis Benchtop 3 l) and allowed 12 h to dry (i.e. lyophilization). The so-synthesized hydrous ACMC was stored in a desiccator with silica gel (rH = 3%) to prevent its transformation to crystalline CaCO_3_. This material is designated as ‘hydrous ACMC’. In a subsequent step, a portion of the freeze-dried ACMC was further dried in a horizontal Netzsch DIL 402 C Dilatometer furnace at 250°C in a dynamic high-purity N_2_ atmosphere (flow rate 100 ml min^−1^; purity >99.999%) for up to 6 h. This material will be designated here as ‘anhydrous ACMC’.

### (b) Powder X-ray diffraction

The hydrous ACMC was analysed by X-ray diffraction using a PANalytical X'Pert Pro diffractometer (Co-K*α* radiation) at a 2*θ* range from 4 to 85° and a scan speed of 0.03° s^−1^ resulting in a total measurement time of 45 min. The anhydrous ACMC was measured on a Seifert 3003TT Bragg-Brentano focusing diffractometer with Cu-Anode X-ray tube operated at 40 kV, 40 mA, incident beam Ge (111) monochromator (Huber) selecting the Cu-K*α*_1_ wavelength, and a linear Meteor (Dectris) photon-counting detector. Total measurement time from 5.0° 2*θ* to 80°2*θ* was 210 min.

### (c) Transmission electron microscopy

High-angle annular dark-field scanning transmission electron microscopy (HAADF-STEM) images, electron diffraction patterns, energy-dispersive X-ray (EDX) spectra and elemental mappings were obtained in a probe-corrected FEI Themis microscope operated at an acceleration voltage of 300 kV and equipped with a SuperXG1 EDX detector. Hydrous and anhydrous materials were deposited by gently diving the grid (2 nm carbon-coated QUANTIFOIL TEM grid) into powder samples. The STEM images were analysed with the DigitalMicrograph Gatan Microscopy Suite 3 software (Gatan Inc., v. 3.41.2938.1). EDX spectra and elemental mapping images were acquired and evaluated with the Velox Software (ThermoFisherScientific).

### (d) Fast scanning differential scanning calorimetry (FDSC)

#### (i) Determination of the glass transition temperature

FDSC measurements were performed using a Mettler-Toledo Flash DSC 2+ operated with UFS 1 sensors, described in [7]. The sample support temperature in the calorimeter was set to 29°C and the sensor was purged with high-purity N_2_ at a flow rate of 30 ml min^−1^. To improve the thermal contact between sample and sensor the active zone of the sample side of the sensor was wetted with a very small amount of silicon oil (AK 500.000 Wacker). To create a thin silicon oil film, the sensor was heated five times at 2000 K s^−1^ to 250°C without sample. The sample was placed on the active zone of the sensor using a hair with a fine tip. Unless otherwise noted, the hydrous ACMC was dried in an external furnace at 250°C for 2 h. Before the heating measurement the sample was additionally dried at 250°C for 60 s or 600 s on the FDSC sensor. The sample mass was between 60 and 130 ng. The sample mass was estimated from the crystallization enthalpy of Δ*h*_c_ = 156.6 J g^−1^ measured with conventional DSC. This method is described in [8]. Samples were measured between 30°C and 520°C with heating rates between 2000 and 6000 K s^−1^.

#### (ii) Determination of the crystallization kinetics

Due to temperature limitations (max. T < 520°C) of the UFS 1 sensors, the crystal-growth kinetics has been measured by using a UFH 1 sensor. The sample support temperature in the calorimeter was set to −80°C using a Huber intracooler TC100, and the sensor was purged with high-purity CO_2_ (to prevent thermal decomposition) at a flow rate of 60 ml min^−1^. We followed the same procedure as described above to improve thermal contact. Before the measurement the sample was dried at 250°C for 60 s on the FDSC sensor. The sample mass was between 20 and 40 ng (see [8] for method). Dried samples were measured between 50°C and 580°C with heating rates of 2000, 4000 and 5000 K s^−1^.

### (e) Thermal gravimetry/conventional differential scanning calorimetry (TGA/DSC)

Conventional thermal gravimetry combined with differential scanning calorimetry has been performed with a Mettler-Toledo TGA/DSC 3 + equipped with a XP1U balance. The hydrous and anhydrous samples (3–5 mg) have been heated in Pt-crucibles with a lid up to 400°C in a dynamic, high-purity N_2_ atmosphere (100 ml min^−1^ flow rate).

## 3. Results

### (a) Chemical and morphological characterization

**Figure 1.**
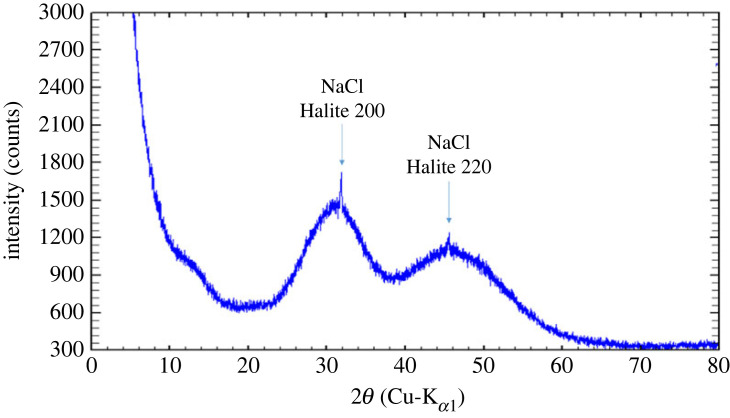
XRD pattern of anhydrous ACMC. The two visible sharp diffraction peaks are due to a very small impurity of NaCl (less than 0.5 at.%) in the material. (Online version in colour.)

The absence of crystalline phases (except for impurities, NaCl) in the ACMC was confirmed by X-ray diffraction (figure 1). The NaCl impurities are due to the synthesis with Na- and Cl-bearing chemicals. Not all NaCl can be washed out as prolonged exposure to water will lead to crystallization of the ACMC.

High-angle annular dark-field scanning transmission electron microscopy (HAADF-STEM), electron diffraction, and energy-dispersive X-ray (EDX) spectroscopy were used to analyse the morphology, size, structure and composition of ACMC particles. To avoid any artifacts introduced by the TEM sample preparation (incl. recrystallization and dehydration of particles in water or/and alcohol containing solution, ion-beam damage, etc.) the hydrous and anhydrous powder samples were mechanically transferred onto TEM grids.

**Figure 2.**
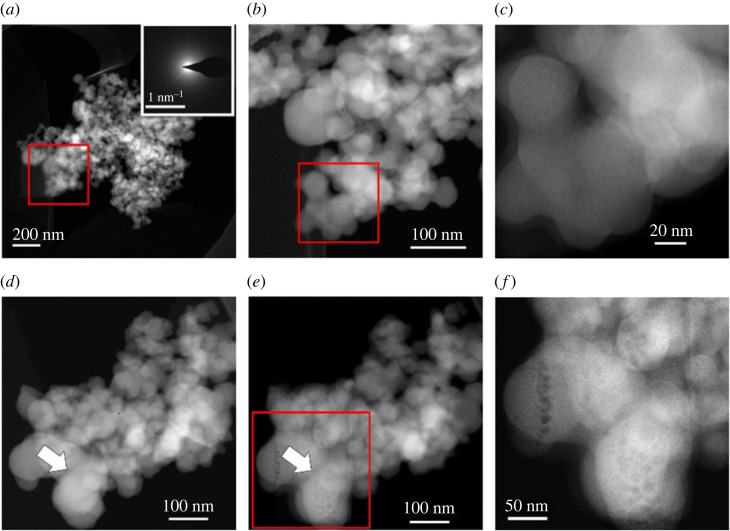
HAADF-STEM images of hydrous ACMC sample. (*a–c*) Series of images with increasing magnification. Inset in (*a*) shows the electron diffraction pattern (representative for amorphous material) recorded from these aggregates. (*b*) is a magnification of (*a*, red square) and (*c*) is a magnification of (*b*, red square). (*d*) and (*e*) subsequent transformation of particles under electron beam. White arrows point at visible beam damage. (*f*) Enlarged image of electron-beam damaged porous ACMC particles (position of red frame in (*e*)). (Online version in colour.)

**Figure 3.**
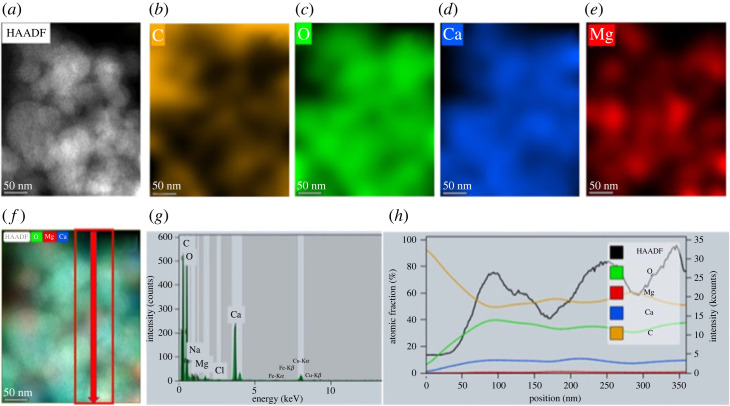
HAADF-STEM image and EDS analysis of a hydrous ACMC sample. (*a*) HAADF-STEM image. (*b–e*) Elemental mapping images showing distribution of (*b*) – carbon, (*c*) – oxygen, (*d*) – calcium, (*e*) – magnesium. (*f*) Overlay-image of HAADF and distribution maps of oxygen, calcium and magnesium. (*g*) EDX spectrum. (*h*) Compositional profile showing distribution of elements (O, C, Mg, Ca) and variation of HAADF-STEM contrast within the area indicated in (*f*). Although the HAADF-STEM contrast indicates strong variation in sample thickness through the profile, the compositional ratio of main elements stays the same. Note: the presence of sodium and chlorine traces (less than 0.5 at.%) is consistent with XRD measurements showing the presence of NaCl. The presence of Fe and Cu peaks in EDX spectrum are common artifacts from objective-lens pole pieces and TEM grid. (Online version in colour.)

HAADF-STEM images of the hydrous sample show random aggregates of polydisperse spherical particles with diameters ranging from approximately 20 nm to approximately 100 nm (figure 2*a–c*). Electron diffraction (figure 2*a*) and high resolution HAADF-STEM imaging (figure 2*c*) confirm the amorphous nature of all particles in this analysed sample. In contrast to a previous report on amorphous calcium carbonate [11], our data show no experimental evidence of hierarchically-ordered organization of ACMC spherical particles (i.e. aggregates of polydisperse particles composed of monodisperse particles). Energy-dispersive X-ray (EDX) measurements (figure 3) show homogenous distribution of calcium and magnesium ions within amorphous particles (with molar ratio Mg^2+^/(Ca^2+^ + Mg^2+^) = 0.0516 ± 0.011) (average of 616 data points).

**Figure 4.**
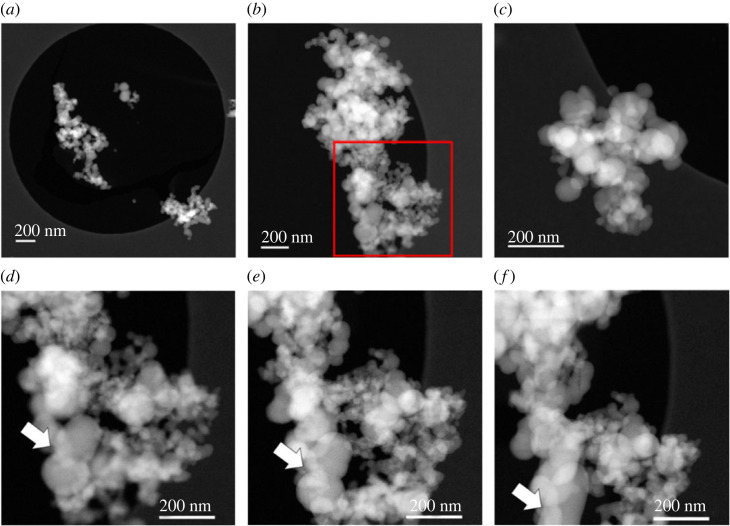
(*a–c*) HAADF-STEM images of anhydrous ACMC on a Quantifoil grid with ultrathin carbon film on a carbon support. (*d*), (*e*) and (*f*) subsequent transformation of particles under electron beam incidence, in the area in (*b*) highlighted in red. White arrow points out deformation and fusion (e.g. sintering) of particles. (Online version in colour.)

**Figure 5.**
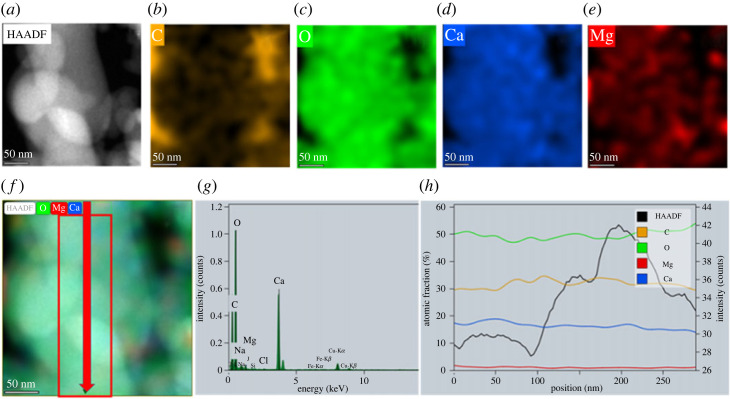
HAADF-STEM image and EDX analysis of anhydrous ACMC. (*a*) HAADF-STEM image. (*b–e*) Exemplary elemental mapping images showing distribution of (*b*) – carbon, (*c*) – oxygen, (*d*) – calcium, (*e*) – magnesium. (*f*) Overlay-image of HAADF and distribution maps of oxygen, calcium and magnesium. (*g*) EDX spectrum. (*h*) Compositional profile showing distribution of elements (O, C, Mg, Ca) and variation of HAADF-STEM contrast within the area indicated in (*f*). (Online version in colour.)

In the case of the anhydrous ACMC sample (250°C, 6 h), the random aggregates (with network-like structure) are composed of distorted spherical-like particles with diameters ranging from approximately 20 nm to approximately 150 nm (figure 4). The slight deviation of particle size and shape compared with the freeze-dried only samples (figure 2) could be explained by dehydration and/or partial fusion (e.g. sintering) of particles during the long drying at higher temperature. Nevertheless, elemental mappings and compositional profiles (figure 5) confirm the homogeneous distribution of calcium and magnesium ions within materials with molar ratio Mg^2^ ^+^/(Ca^2+^ + Mg^2+^) = 0.051 ± 0.021). We note, however, that these two samples behave differently under long exposure to the electron beam. The spherical particles of hydrous samples show, after several minutes, a very porous surface structure (figure 2*d–f*), while the particles of the anhydrous sample rather deform and sinter (figure 4*d–f*). The molar fraction of Mg in ACMC has been determined to be 0.051 ± 0.021 (average of 206 data points).

The water content was estimated using the first loss step (ambient to 250°C) in the TGA curve (following [12]). The hydrous (freeze-dried) sample contained 8.46 ± 0.1 wt% of water (0.509 mole H_2_O per mole ACMC). The pre-dried (250°C, 6 h) sample curve showed no weight loss and can therefore be considered as nominally anhydrous. For amorphous calcium carbonate (ACC) the release of minor amounts of water upon heating to 400°C was detected [13], but it was concluded that samples heated to 200°C generated ‘near anhydrous’ ACC under extended isothermal conditions.

The enthalpy of crystallization for the anhydrous ACMC (as mentioned above) is of Δ*h*_c_ = 156.6 ± 9 J g^−1^. This is within the errors of the enthalpy of crystallization for anhydrous ACC to calcite [12].

### (b) Fast scanning differential scanning calorimetry (FDSC)

#### (i) Thermal behaviour of differently dried samples

Due to a broad endothermal peak caused by water loss, which is superimposed on the thermal effects, the heat flow curves of hydrous samples could not be used for further analyses. Therefore, the material was dried before measurement. To investigate the effect of remaining water on the glass transition and the crystallization kinetics of ACMC, different drying protocols were applied. The following procedures were used:

(A) A hydrous sample (hydrous ACMC) was placed on the sensor and heated to 250°C at 2000 K s^−1^ and annealed for 1 min. The sample was then cooled to 30°C at 2000 K s^−1^.
(B) A previously dried sample (anhydrous ACMC; 2 h at 250°C in an external furnace) was annealed using the same procedure.
(C) A dried sample (anhydrous ACMC; 2 h at 250°C in an external furnace) was annealed at 250°C for 10 min.
(D) The annealing procedure of (C) was extended to 1 h.

**Figure 6.**
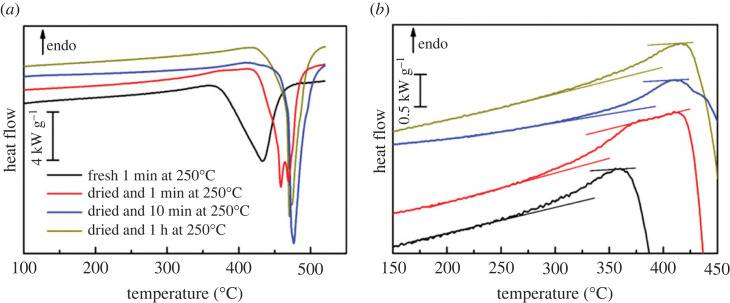
DSC curves of the differently dried samples (‘fresh’ denotes hydrous ACMC; ‘dried’ denotes anhydrous ACMC). The curves are shifted vertically. (*a*) Crystallization behaviour. (*b*) Glass transition range. (Online version in colour.)

Immediately after this annealing, the sample was heated twice at 2000 K s^−1^ to 520°C, and in between, the sample was cooled at 2000 K s^−1^. All treatments were performed in a dynamic high-purity N_2_ atmosphere. The results (i.e. heat flow curves) of the first heating are displayed in figure 6*a*. During heating, prior to the exothermal crystallization peaks, the DSC curves display a small endothermic signal. The crystallization peak shifts to higher temperature with longer annealing time at 250°C. This indicates that the remaining water accelerates the crystallization process. When using previously dried samples, the crystallization peak has a complex shape with multiple shoulders or double peaks, indicating a more complex crystallization behaviour. The samples are clusters of individual particles that crystallize individually. The second heating curves show no thermal effects, as expected for crystallized material.

To examine the behaviour before crystallization in more detail, the curves in figure 6*a* were enlarged (figure 6*b*). Prior to the crystallization peak, the curves show a rise typical of that exhibited in a glass transition. At the end of this glass transition, the molecular mobility is high enough for crystallization to proceed. The dried sample annealed at 250°C for 1 min exhibits an extended temperature interval of approximately 50°C with a linear section between the end of the glass transition and the onset of the crystallization peak, which is absent in the other heat flow curves.

The tangents in the glassy and the liquid state (figure 6*b*) are used to determine the glass transition. The glass transition temperatures, *T*_g_, and the temperatures associated with the first maxima of the crystallization peak, *T*_c_, are listed in table 1. The glass transition temperature is determined as the limiting fictive temperature [14,15].

**Table 1.** Glass transition temperature, *T*_g_, and crystallization peak temperature, *T*_c_, for differently dried samples measured during heating at 2000 K s^−1^.

sample	*T*_g_ (°C)	*T*_c_ (°C)
hydrous ACMC, 1 min at 250°C	339.7	432.6
anhydrous ACMC, 1 min at 250°C	357.4	458.6
anhydrous ACMC, 10 min at 250°C	388.0	476.8
anhydrous ACMC, 1 h at 250°C	388.2	470.8

The data shows that the previously dried sample absorbs water from the atmosphere during the preparation procedure. This water can be removed during a relatively short annealing at 250°C. The water is widely removed after annealing of 10 min. Longer annealing has practically no effect on the glass transition and crystallization.

The high temperature required for sample dehydration indicates that the water in ACMC is structurally bound. The remaining water strongly affects the *T*_g_ and consequently the molecular mobility in ACMC. This may be the origin of differences in crystallization kinetics.

#### (ii) Determination of the glass transition temperature

To determine the properties of anhydrous ACMC, previously dried material was heated to 250°C for 10 min to remove water, which was absorbed during the preparation process.

**Figure 7.**
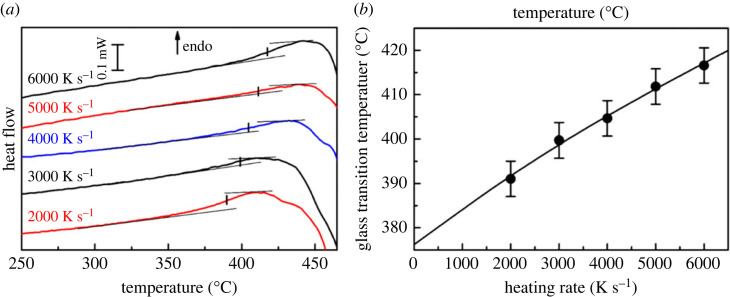
Glass transition of anhydrous ACMC measured at different heating rates. (*a*) Heat flow curves. (*b*) The measured glass transition temperature versus the heating rate. The data are fitted with equation (1). (Online version in colour.)

Samples were heated with heating rates between 2000 K s^−1^ and 6000 K s^−1^. Larger samples were used for slower heating rate measurements. The heat flow curves in the glass transition range are shown in figure 7*a*. Tangents were used to localize the glass transition range and a short vertical line indicates the glass transition temperatures. All curves exhibit a single glass transition which shifts to higher temperature with increasing heating rate.

As all samples have the same thermal history, the glass transition temperature should be solely a function of the heating rate [16]. The measured dependency on the heating rate is a consequence of the influence of thermal inertia of the calorimeter and the sample that increase the temperature and the width of the glass transition [17,18]. A simplified model of this effect on the glass transition temperature is discussed in [16]. The heating rate dependency of *T*_g_ follows

3.1 $$T_{\text{g}}=T_{\text{g}0}+\beta \tau \left(1-\exp\left(-\frac{T_{\text{g}0}-T_{0}}{\beta \tau }-1\right)\right),$$

where *β* is the heating rate, *τ* the thermal lag due to sample and instrument, *T*_0_ the extrapolated temperature of the first indication of the glass transition and *T*_g0_ is the extrapolated glass transition temperature.

The glass transition temperatures as a function of the heating rate are plotted in figure 7*b*. The data are fitted with equation (3.1) using the fit parameters *T*_0_ = 329°C and *τ* = 7.9 ms and *T*_g0_ = 376°C. The time constant corresponds to the expected thermal lag. The glass transition temperature of the investigated glass is approximately 376°C and the intensity of the glass transition, the relaxational heat capacity, is Δ*c*_p_ = 0.16 J/(g K).

#### (iii) Determination of the crystallization kinetics

The effect of the thermal inertia of the instrument and sample affect the FDSC curves. For kinetic studies, the effects of such smearing of the curves must be investigated and considered. A simple de-smearing procedure is based on a one-parameter heat flow model. Knowing the time constant of the heat transfer, *τ*, the measured heat flow, *Φ*_m_ can be corrected by using the time derivative [19,20]

3.2 $$\Phi_{\text{c}}=\Phi_{\text{m}}+\tau \frac{\text{d}\Phi_{\text{m}}}{\text{d}t}.$$

**Figure 8.**
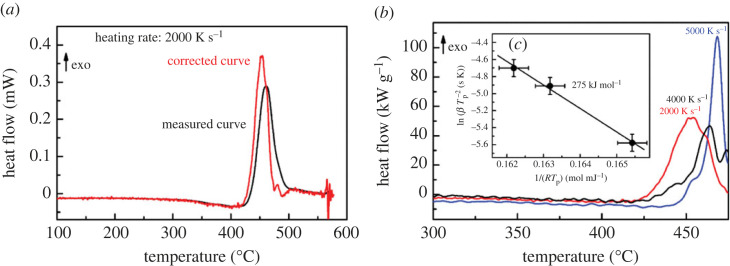
(*a*) Measured and corrected heat flow curves measured at 2000 K s^−1^ (UFH-1 sensor). (*b*) Peak of the primary crystallization of the corrected heat flow curves. (*c*) Kissinger plot for determination of the activation energy of the primary crystallization. (Online version in colour.)

The time constant *τ* = 5 ms is determined from the heating rate dependence of the onset of the glass transition. The influence of the correction on the FDSC curves is shown in figure 8*a*. For the heating rate of 2000 K s^−1^, the peak temperature is decreased by about 10°C. The corrected peaks of the primary crystallization for the three heating rates used are displayed in figure 8*b*. Due to the increased noise in the corrected curves, a detailed evaluation of the glass transition is not possible. Measurements at higher rates were not possible due to the limited thermal contact between sample and sensor.

The heating rate dependence of the peak temperature of the corrected crystallization peaks is used to determine the activation energy. For this purpose, the Kissinger plot [21,22] was used (figure 8*c*). The activation energy of 275 kJ mol^−1^ is the slope in this diagram.

## 4. Discussion

We have shown that amorphous, anhydrous ACMC is a structural glass. The identification of a glass transition using fast scanning rates prior to crystallization confirms the transition between a non-ergodic amorphous form and an equilibrium ergodic state with diffusion timescales sufficiently fast to allow crystallization. This is consistent with the broad definition of a glass transition [5] and relaxation of structural states within the amorphous structure. The materials produced are homogeneous, amorphous solids forming random aggregates and confirm that it is possible to produce glasses avoiding the liquid state entirely. The apparent temperature-dependence of the glass transition on scanning rate is shown to be a result of the thermal lag, allowing a glass transition temperature to be defined, a reflection of the anhydrous, amorphous structure produced by the freeze-drying (lyophilization) step.

A recent study of the glass transition of recovered K-Mg carbonate produced at high pressure [23] shows a glass transition onset at 225°C and exothermic crystallization peaks at ∼330 and 340°C. This is clearly a structural glass in the sense of [5], produced by quenching a liquid. Furthermore, the K–Mg carbonate glass can contain water which influences the distortion of carbonate units and (see Weidendorfer *et al*. [1]) the onset of glass transition. When comparing the glass transition and derived viscosity/structural relaxation behaviour in K–Mg and Na-carbonates with that for ACMC there is no obvious distinction between the structural relaxation of the liquid and liquid-derived carbonates and the dehydrated amorphous precipitate and therefore, no distinction between their glassy properties.

The present study demonstrates a methodology that includes carefully controlled dehydration steps and FDSC which can be applied more extensively to amorphous carbonate and hydroxycarbonate systems. In the present contribution we have focused on ACC containing small amounts of Mg^2+^ which is justified partly because biogenetically produced amorphous calcium carbonate contains small amounts of magnesium (see [12]) but more practically because the corresponding ACMC is more stable and less prone to hydration/crystallization. By carefully controlling water content through dehydration steps, the glass transition for the anhydrous amorphous forms can be identified. The presence of water within carbonate systems determines the glass transition temperature and crystallization behaviour and further studies using the techniques outlined here will allow a more detailed investigation of biogenically ACCs. Specifically, to identify the role of water in defining different glassy types, the interplay between water content and crystallization and the role of magnesium and other impurities in determining the relaxation of structural entities and crystallization kinetics.

There are other amorphous forms of carbonate, specifically Cu–Zn oxycarbonates such as synthetic georgeite (see [24]) that are of interest as precursors to Cu–Zn catalysts which are ideal systems for FDSC studies. The formation of these amorphous materials and crystallization of the Cu–Zn nanoparticles during dehydration and calcination steps may also include relaxation of hydrous glasses and formation of intermediate amorphous forms that will influence the crystallization history. The FDSC studies also provide the framework to explore the differences in structural relaxation of amorphous carbonate produced by different synthesis routes including hydrothermal routes and to compare crystallization pathways.

The present study demonstrates that ACMC, hitherto assumed to be an amorphous solid, has a calorimetric glass transition and is indistinguishable from a structural glass. As such ACMC can be included within the large family of glasses produced by different routes (cf. glasses by evaporation or electrodeposition) and the glass transition also represents a relaxation of structure (as enthalpy) from a non-ergodic to ergodic regime. Although it cannot be concluded that all materials considered amorphous solids show these characteristically glassy features this study does indicate that a glass transition in amorphous materials can be identified if ultrafast thermal analysis is applied.

## Data Availability

Data is accessible through the following link: https://doi.org/10.5281/zenodo.7638928 [25].
